# Technology in Aging

**DOI:** 10.1093/geroni/igaf122.1791

**Published:** 2025-12-31

## Abstract

Digital tools in Aging including telehealth, smart phones and video capture research are described.

